# Imaging Tasks Scheduling for High-Altitude Airship in Emergency Condition Based on Energy-Aware Strategy

**DOI:** 10.1155/2013/242836

**Published:** 2013-06-22

**Authors:** Li Zhimeng, He Chuan, Qiu Dishan, Liu Jin, Ma Manhao

**Affiliations:** Science and Technology on Information Systems Engineering Laboratory, National University of Defense Technology, Changsha 410073, China

## Abstract

Aiming to the imaging tasks scheduling problem on high-altitude airship in emergency condition, the programming models are constructed by analyzing the main constraints, which take the maximum task benefit and the minimum energy consumption as two optimization objectives. Firstly, the hierarchy architecture is adopted to convert this scheduling problem into three subproblems, that is, the task ranking, value task detecting, and energy conservation optimization. Then, the algorithms are designed for the sub-problems, and the solving results are corresponding to feasible solution, efficient solution, and optimization solution of original problem, respectively. This paper makes detailed introduction to the energy-aware optimization strategy, which can rationally adjust airship's cruising speed based on the distribution of task's deadline, so as to decrease the total energy consumption caused by cruising activities. Finally, the application results and comparison analysis show that the proposed strategy and algorithm are effective and feasible.

## 1. Introduction

One of the most significant features of emergency scheduling problem (ESP) is timeliness; that is, the execution of task must be completed in its deadline. Otherwise, the task will lose its executive value or become invalid [1–3]. Under the emergency condition, the imaging task has its observation slot to reflect the requirements on the execution timing interval. For example, the emergency tasks, such as the observations on the targets about moving missile system, massing troops, and cruising battleship, generally need the responding agencies to scout timely in order to rapidly analyze the situation and to plan the operational activity.

Over the last decade, many military groups such as the US army have been devoted to development of the emergency imaging technology and improvement of the quick response ability of the reconnaissance system by incorporating multiple platforms. High-altitude airship is a promising solution for the emergency observation platform in the near-space [4, 5]. Unlike conventional heavier-than-air (HTA) aircraft, high-altitude airship is a lighter-than-air (LTA) aircraft equipped with steering and propulsion systems, and it generates lift force through the buoyancy instead of aerodynamics [6]. At present, high-altitude airship located in the near-space has attracted wide attention in many countries, and it is well known that some projects have been studied, for example, the HARV and HAA projects [7] in USA, Sky cat and CL-160 projects [8] in the European Union, ETRI [9] in South Korea, and Sky Net in Japan [10]. The scientists and engineers in China have conducted corresponding researches since the last century, and the verification airship has completed its low-altitude flight experiment in 2003.

As a new application platform, the high-altitude airship has many advantages in reconnaissance activities. For instance, it has a long duration, and a great deal of load carrying, and can achieve the fixed-point successive observation, and so forth [11]. In comparison with the traditional unmanned aircraft vehicle (UAV) [12, 13], the high-altitude airship can be operated continuously for several months, even for more than one year in the assigned airspace. It is also easy to acquire data and information uninterruptedly in a long period. Due to the fuel restriction, UAV has to implement the aerial refueling or return to the base frequently, so it is impossible to achieve the long-term and continuous monitoring at a lower cost. The capsule of the high-altitude airship is usually made from the nonmetallic materials with less electromagnetism and heat reflecting, which makes it hard to be captured by radar. In addition, the high-altitude airship is invulnerable to be attacked and intercepted by many air-defense missiles, due to the operational height which is out of their fire range. Compared with the imaging reconnaissance satellite [14–16], the high-altitude airship has stronger ability of rapid response. Generally speaking, the ground support equipments for launching a high-altitude airship are fewer in number and have shorter period of launching preparation. Therefore, the theater reconnaissance, surveillance, and warning system can be established by high-altitude airship in a few hours, and the mass deployment can be rapidly implemented with its strong maneuverability. In terms of the efficiency-cost ratio, the in-orbit time of a high-altitude airship is nearly equal with that of an imaging reconnaissance satellite, but the usage cost is far less than the latter. In addition, the satellite is restricted by the fixed orbit in use and only can observe the targets in a certain time slot. On the contrary, the high-altitude airship can achieve the long-term and continuous observation on the target in the hover-and-stare way. Due to the previous advantages, the high-altitude airship has huge application potential in the emergency activities, such as the antiterrorism, disaster relief, and regional battles. The aforementioned advantages have turned high-altitude airship into an ideal imaging observation platform.

Existing studies on high-altitude airship are scattered over a range of journals, conferences, books, and reports. Rao et al. [17] presented a mission path following controller for the airship by employing artificial neural network (ANN). Tan et al. [18] introduce some methods and techniques to realize lightweight structure and present a review of current research on high-altitude airship with lightweight structures. Bessert and Frederich [19] investigated the aerodynamics behavior of high-altitude airship and presented a novel technology to test the aerodynamics on the structural behavior of airship. Ren et al. [20] analyzed the aerodynamics problems of high-altitude airship while launching, recovering, hovering, and introducing the achievement of airship dynamics research. Especially, there are numerous studies on energy system of high-altitude airship. Wang et al. [21] presented a novel computation method for solar radiation on solar cells of the airship, given the effect of the airship's attitude on the performance of its energy system. Ma and Sun [22] developed a power management framework of high-altitude airship, which can rationally distribute power to subsystems so as to lighten the energy consumption in certain situation. Wang et al. [23] proposed an energy balance method to analyze the regenerative energy system, which can streamline the configuration design of high-altitude airship. In addition, there are also great deal of works focusing on the propulsion system. Chen et al. [24] constructed a simulation model and made some analyses about propeller of high-altitude airship. Jordi et al. [25] discussed the biomimetic principles for the structural design of airship. Various development tests are completed in their research, including wind tunnel testing and flight trials. Unfortunately, there are only a handful of works reported to date in the literature that propose the task planning of high-altitude airship, which greatly degrades the system performance such as task guarantee ratio and energy consumption.

In this paper, we focus on the imaging tasks scheduling problem on the high-altitude airship under the emergency condition. The power-speed model is constructed, which is employed to evaluate the energy consumption during the airship's reconnaissance actives. We convert this scheduling problem to constrain satisfaction problem (CSP), then a heuristic algorithm based on the optimization sequence rule (OSR) is presented to obtain the task ranking scheme, and a value task detecting (VTD) method is provided to detect the key nodes that each airship needs to fly through in sequence. An energy-aware strategy (EAS) is also provided to optimize the task planning by rationally adjusting the cruising speed of airship. The simulation results show the effectiveness of this strategy.

The reminder of this paper is structured as follows.  Section 2 makes detailed description on the reconnaissance process of high-altitude airship and establishes the corresponding models and proposed optimization objects.  Section 3 converts the original problem into three sub-problems by adopting hierarchy architecture and design the solution algorithms, respectively. The simulation experiments and performance analysis are given in Section 4. The final section will conclude this paper and discuss the future research direction.

## 2. Problem Description and Modeling

The application of high-altitude airship in imaging reconnaissance activity is an asset for other reconnaissance equipments, and it is of great significance to build and improve the reconnaissance network. To facilitate analysis and modeling of this problem, we summarize the main notations used throughout this paper as follows:

 
*T*_*p*_ = [*t*_start_, *t*_end_]: the active period of airship, where *t*_start_ refers to the starting time and *t*_end_ refers to the completion time of observation activity;
 
*Task* = {*task*_1_, *task*_2_,…, *task*_*n*_}: the imaging task set, where the element *task*_*i*_ refers to the *i*th task, and *n* refers to the task number;
 
*sit*_*i*_ = (*x*_*i*_, *y*_*i*_): the observation projection position of  *task*_*i*_, where *x*_*i*_, *y*_*i*_ denote the horizontal coordinate and vertical coordinate, respectively;
 
*sit*_0_ = (*x*_0_, *y*_0_): the projection coordinate of airship at the beginning;
 
*S*_max⁡_: the maximum cruising speed of airship;
 
*td*_*i*_: the deadline of *task*_*i*_;
 
*tb*_*i*_: the beginning timing instant of *task*_*i*_;
 
*te*_*i*_: the completion timing instant of *task*_*i*_;
 
*t*_*i*_: the duration time of *task*_*i*_, which includes the system stability time, load switch time, and data storage time;
 
*S*_*i*,*j*_: the average cruising speed of airship between *sit*_*i*_ and *sit*_*j*_;
 
*d*_*i*,*j*_: the distance between *sit*_*i*_ and *sit*_*j*_;
 
*en*_*i*,*j*_^active^: the energy consumption of cruising from *sit*_*i*_ and *sit*_*j*_;
 
*en*_*i*_^static^: the energy consumption of balance resistance while airship in fixed-point state.

### 2.1. The Process of Task Execution

The imaging payload is usually installed in the cabin of high-altitude airship, which can be tilted or rotated within a certain angle to observe targets on the ground. During the task execution, the high-altitude airship flies according to the predetermined route and hover at a certain observation position, and in this way, the targets can be observed by imaging payload.

**Figure 1:**
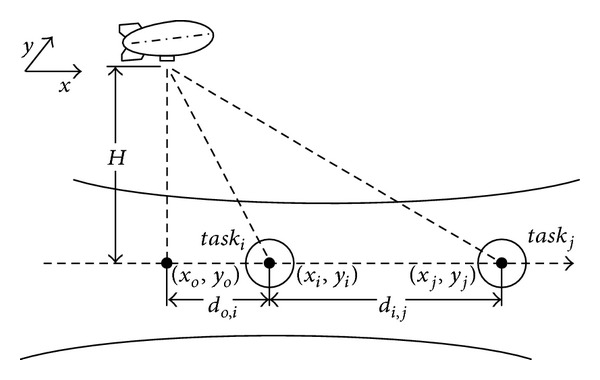
The cruising of high-altitude airship.

As shown in Figure 1, *task*_*i*_ and *task*_*j*_ are located in different positions. After completing *task*_*i*_, the airship moves to another observing position to execute *task*_*j*_. The cruising of airship will take a long time due to the limited speed, which makes it almost impossible to execute *task*_*j*_ timely.

**Theorem 1**

If *task*_*i*_ can be observed before its deadline, it is called a value task; otherwise, it is called an invalid task.

Assume that the current time is *T*_0_, and *L* is the distance between the airship and *task*_*i*_. If *task*_*i*_ is a value task, the following conditions must be met:

(1) $$\begin{array}{c} T_{0}+\frac{L}{S_{\max}}+t_{i}\leq td_{i}. \end{array}$$

Obviously, the number of value tasks decreases with the time advancement, and this trend is irreversible. Considering that the imaging targets are widely distributed in the battle area, it is nearly impossible to ensure all tasks to be observed timely. Therefore, it is necessary to choose a reasonable task set and allocate the observation time for airship in accordance with various constraint conditions, so as to realize maximum efficiency of observing activity.

### 2.2. The Power-Velocity Model

The energy system of high-altitude airship converts the solar radiation into electrical energy, thereby providing energy to the entire platform. Assume that the power of the propulsion system is *P*_*t*_, and the efficiency is *η*; then, the actual propulsion power is

(2) $$\begin{array}{c} P_{r}=P_{t}\times \eta . \end{array}$$

**Figure 2:**
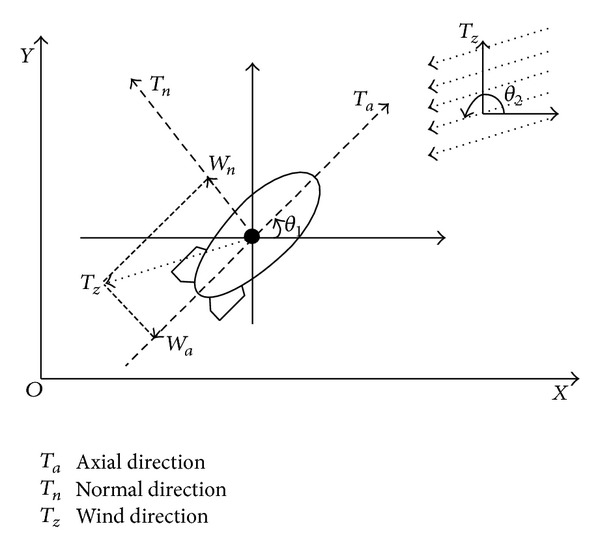
The plane motion of high-altitude airship.

The cruising of high-altitude airship is subject to the impact of wind. The magnitude and direction of the propeller thrust are adjusted to balance the wind resistance, so as to realize continuous monitoring at fixed-point position. In this paper, it is assumed that the airship mainly relies on the electric propeller device to provide the thrust, which can be quickly adjusted in accordance with the wind direction and task position [26]. Since the working height of the airship is maintained, we only need to consider its horizontal movement as shown in Figure 2.

Normally, the wind field in near-space is stable; hence, the wind speed can be decomposed along the axial direction and normal direction of the airship. Consider

(3) $$\begin{array}{c} W_{a}=W\cos\left(\theta_{2}-\theta_{1}\right),\\ W_{n}=W\sin\left(\theta_{2}-\theta_{1}\right), \end{array}$$

where *W* is the velocity of wind, *θ*_1_ is the cruising direction of airship, and *θ*_2_ is the direction of wind field.

The typical power-velocity model of airship is [24, 27, 28]:

(4) $$\begin{array}{c} P_{a}=\frac{1}{2}\rho V^{2/3}{\left(S-W_{a}\right)}^{3}C_{a}\\ P_{n}=\frac{1}{2}\rho V^{2/3}W_{n}^{3}C_{n}, \end{array}$$

where *ρ* is the air density, *V* ^2/3^ is the characteristic area of airship based on its volume *V*, *S* is the airship's cruising speed relative to the ground, and *C*_*a*_ and *C*_*n*_ refer to the aerodynamic coefficients.

### 2.3. The Optimization Objectives

Let *X* = (*x*_1_, *x*_2_,…, *x*_*n*_), *Y* = [*y*_*i*,*j*_]_*n*×*n*_, and *S* = [*S*_*i*,*j*_]_(*n*+1)×(*n*+1)_ be the decision variables. If *task*_*i*_ is effectively performed, then it is a value task (assume that *x*_*i*_ = 1); otherwise, let *x*_*i*_ = 0. If *x*_*i*_ *x*_*j*_ = 1 and *task*_*i*_ is the preceding task of *task*_*j*_, then let *y*_*i*,*j*_ = 1; otherwise, let *y*_*i*,*j*_ = 0.

The primary optimization objective of the tasks scheduling problem on the high-altitude airship is to maximize the guarantee ration *ER*(*X*, *Y*, *S*):

(5) $$\begin{array}{c} ER\left(X,Y,S\right)=\frac{1}{n}\sum_{i=1}^{n}x_{i}. \end{array}$$

The total energy consumption *En*_total_  (*X*, *Y*, *S*) should be minimized on the base of the maximization of *ER*(*X*, *Y*, *S*). *En*_total_(*X*, *Y*, *S*) includes two parts; *En*_active_(*X*, *Y*, *S*) and *En*_static_(*X*, *Y*, *S*) are the energy consumptions caused by cruising and balance resistance in the suspension position, respectively.

According to (2)–(4), the energy consumption of airship's cruising from *sit*_*i*_ to *sit*_*j*_ is

(6) eni,jactive=ρV2/3Cndi,j2ηSi,j|Wsin(θ2−θ1)|3+ρV2/3Cadi,j2ηSi,j|Si,j−Wcos⁡(θ2−θ1)|3.

If the high-altitude airship locate in the observation position of *task*_*i*_, the energy consumption caused by resisting the effect of wind is

(7) enistatic=ρV2/3Cati2η|Wcos⁡(θ2−θ1)|3+ρV2/3Cnti2η|Wsin(θ2−θ1)|3.

According to (6) and (7), the total energy consumption of airship during execution of the tasks is

(8) Entotal(X,Y,S)=Enactive(X,Y,S)+Enstatic(X,Y,S)=∑i=1 n∑j=1nyi,jeni,jactive+∑i=1nxienistatic.

### 2.4. The Programming Model

In the typical route planning of UAV, it is necessary to simultaneously consider the maximum turning angle, maximum climbing angle, minimum flight altitude, minimum path length, and other constraints. The purpose is to ensure that the cruising path can meet the aircraft's maneuvering characteristics and reduce the probability of damaging the aircraft in the no-fly zone and threatened area. As for the high-altitude airship in this paper, its working space has no spatial constraint, so the no-fly zone is an unnecessary consideration. At the same time, the low speed and slow dynamics provide the airship with a large-angle cornering ability, and it is also unnecessary to consider the minimum path length due to its suspension ability. Moreover, the threatened area of the high-altitude airship can be ignored due to the difficulty of being captured by the radar. However, the main constraints of tasks scheduling on the high-altitude airship are listed as follows. 

*Constraint 1.* The high-altitude airship only executes the observation task within its active period.

*Constraint 2.* Each task can be executed only once, and it must be completed before its deadline. 

*Constraint 3.* If a task can be executed, the execution time should be no less than the required continuous working time. 

*Constraint 4.* Only one preceding task or one following task of each task is allowable at most. 

*Constraint 5.* The preemptive service in the task execution is prohibited. Once the execution starts, the process cannot be terminated until completion. 

*Constraint 6.* Before performing a new task, the airship needs sufficient time to change the observation position. 

*Constraint 7.* For any two tasks to be executed, the certain priority order exists. 

*Constraint 8.* The moving processes of airship only exist in different observation positions. 

*Constraint 9.* The cruising speed of high-altitude airship in each path segment cannot be higher than the maximum cruising speed. 

Let *R*_1_, *R*_2_, and *R*_2_ be the feasible solution space of the decision variables *X*, *Y*, and *S*. In the separate optimization of *ER*(*X*, *Y*, *S*), their optimal solution spaces are *Q*_1_, *Q*_2_, and *Q*_3_, respectively. The programming model of this scheduling problem is given as follows:

(9) Z1(X,Y,S)=max⁡  X∈R1,Y∈R2,S∈R3{1n∑i=1nxi},Z2(X,Y,S)  =min⁡  X∈Q1,Y∈Q2,S∈Q3{∑i=1n∑j=1nyi,jeni,jactive+∑i=1nxienistatic} s.t.{[tbi,tei]⊂[tstart,tend]∑i=1nxi≤1, tei≤tditei−tbi≥ti, if  xi=1∑i=1nyi,j≤1, ∑j=1nyi,j≤1[tbi,tei]∩[tbj,tej]=∅, if  xi·xj=1tei+di,jSi,j−K(1−yi,j)≤tbjyi,j·yj,i=0∑i=1nyi,i=0Si,j≤Smax⁡xi,yi,j∈{0,1}∀i,j∈{1,2,…,m}, k∈{1,2,…,n},

where the former nine inequality formulas correspond to the aforementioned constraints, respectively, and the tenth inequality formula restricts the range of the decision variables.

## 3. Scheduling Algorithms

There exist numerous constraints in the tasks scheduling problem, so it is difficult to solve this problem directly. Therefore, the hierarchical optimization can be used to convert the original problem to the following sub-problems.

1. Determine the priority execution order of the tasks, that is, the task sorting problem, which can be solved by the OSR algorithm.
2. Select the observation tasks for the airship, that is, the value tasks detection problem, which can be solved by the VTD algorithm.
3. Adjust the planning scheme to reduce energy consumption of the airship, that is, the energy conservation problem, which can be solved by the EAS algorithm.

Firstly, the task guarantee ration is the primary optimization objective of this scheduling problem. Thus, this paper considers that maximum cruising speed should be used by the airship to execute tasks as frequently as possible. Then, the distributions of value tasks are tested, and the initial scheduling scheme of the original problem is obtained. On this basis, energy conservation is regarded as another optimization goal. The cruising speed of airship at each leg is adjusted, and the execution time of all value tasks is updated.

**Theorem 2**

The feasible solution of the original problem is expressed with two-tuples *SP* = 〈*vq*, *sq*〉, where *vq* = {*task*_*v*1_, *task*_*v*2_,…, *task*_*vn*_} is the task set rearranged by priority execution order and *sq* = {*S*_0,*v*1_, *S*_*v*1,*v*2_,…, *S*_*v*(*n*−1},*vn*_} is the speed set of airship at each leg.

**Theorem 3**

For any feasible solutions *SP*_1_ and *SP*_2_, if *SP*_1_ is superior to *SP*_2_, then *SP*_1_⊳*SP*_2_ or *SP*_2_⊲*SP*_1_; if *SP*_1_ is inferior to *SP*_2_, then *SP*_1_⊲*SP*_2_ or *SP*_2_⊳*SP*_1_; otherwise, *SP*_1_ = *SP*_2_.

The comparison method of any two feasible solutions *SP*_1_ and *SP*_2_ is presented as follow:

 
*SP*_1_⊳*SP*_2_, if  *ER*(*SP*_1_) > *ER*(*SP*_2_),
 
*SP*_1_⊳*SP*_2_, if  *ER*(*SP*_1_) = *ER*(*SP*_2_), *En*_total_(*SP*_1_) < *En*_total_(*SP*_2_),
 
*SP*_1_ = *SP*_2_, if  *ER*(*SP*_1_) = *ER*(*SP*_2_), *En*_total_(*SP*_1_) = *En*_total_(*SP*_2_),
 
*SP*_1_⊲*SP*_2_, otherwise.

**Theorem 4**

For any effective solution *SP*_1_ = 〈*vq*, *sq*〉 and feasible solution *SP*_2_ = 〈vq*, sq*〉, if *SP*_1_⊲*SP*_2_, then *SP*_2_ is an optimal solution of *SP*_1_.

The conventional methods to obtain effective solutions including (a) improving the priority execution order of tasks in order to obtain more value tasks; (b) reducing energy consumption by adjusting the cruising speed of airship on the basis of ensuring the quantity of value tasks. The previous methods are realized by the OSR algorithm and EAS algorithm, respectively.

### 3.1. The OSR Algorithm

The OSR algorithm sorts all elements in *Task* to get the priority execution order of tasks, and the guarantee ration is the primary optimization objective. Although all tasks are ranked, only part of the tasks can be observed timely; that is, the ranking result is only available for value tasks tested by the VTD algorithm, and it shows the observation sequence of executable tasks. The analysis of sorting rules in two tasks is as follows.

Let *Task* = {*task*_1_, *task*_2_} denote the task set, *T*_0_ the current timing instant and *sit*_0_ the initial position of airship. Given two feasible solutions *SP*_1_ = 〈*vq*_1_, *sq*〉 and *SP*_2_ = 〈*vq*_2_, *sq*〉, where *vq*_1_ = {*task*_1_, *task*_2_}, *vq*_2_ = {*task*_2_, *task*_1_} and *sq* = {*S*_max⁡_, *S*_max⁡_}, there is no harm in assuming that *td*_1_ − *d*_0,2_ *S*_max⁡_^−1^ ≥ *td*_2_ − *d*_0,1_ *S*_max⁡_^−1^, and then, the relationship between *ER*(*SP*_1_) and *ER*(*SP*_2_) is discussed as follows.

(a) If *task*_1_ and *task*_2_ are both value tasks in *SP*_1_, then
  (10) $$\begin{array}{c} T_{0}+d_{0,1}S_{\max}^{-1}+t_{1}\leq td_{1},\\ T_{0}+\left(d_{0,1}+d_{1,2}\right)S_{\max}^{-1}+t_{1}+t_{2}\leq td_{2}. \end{array}$$
  In accordance with the assumption, we can get
  (11) $$\begin{array}{c} T_{0}+d_{0,2}S_{\max}^{-1}+t_{2}\leq td_{2},\\ T_{0}+\left(d_{0,2}+d_{\text{2,1}}\right)S_{\max}^{-1}+t_{2}+t_{1}\leq td_{1}. \end{array}$$
  Then, *task*_1_ and *task*_2_ are both value tasks in *SP*_2_; thereby, *ER*(*SP*_1_) = *ER*(*SP*_2_).
(b) As for *SP*_1_, if *task*_1_ is a value task while *task*_2_ is an invalid task, then:
  (12) $$\begin{array}{c} T_{0}+d_{0,1}S_{\max}^{-1}+t_{1}\leq td_{1},\\ T_{0}+\left(d_{0,1}+d_{1,2}\right)S_{\max}^{-1}+t_{1}+t_{2}>td_{2}. \end{array}$$
  As for *SP*_2_, if *task*_2_ is a value task, then *ER*(*SP*_1_) ≤ *ER*(*SP*_2_); if *task*_2_ is an invalid task, we can still ensure that *ER*(*SP*_1_) = *ER*(*SP*_2_) due to *T*_0_ + *d*_0,1_ *S*_max⁡_^−1^ + *t*_1_ ≤ *td*_1_.
(c) As for *SP*_1_, if *task*_1_ is an invalid task while *task*_2_ is a value task, then
  (13) $$\begin{array}{c} T_{0}+d_{0,1}S_{\max}^{-1}+t_{1}>td_{1},\\ T_{0}+d_{0,2}S_{\max}^{-1}+t_{2}\leq td_{2}. \end{array}$$
  As for *SP*_2_, *task*_2_ is a value task; thereby, *ER*(*SP*_1_) ≤ *ER*(*SP*_2_).
(d) If *task*_1_ and *task*_2_ are both invalid tasks in *SP*_1_, then
  (14) $$\begin{array}{c} T_{0}+d_{0,1}S_{\max}^{-1}+t_{1}>td_{1},\\ T_{0}+d_{0,2}S_{\max}^{-1}+t_{2}>td_{2}. \end{array}$$
  There is an equation *ER*(*SP*_1_) = *ER*(*SP*_2_).

If *td*_1_ − *d*_0,2_ *S*_max⁡_^−1^ ≤ *td*_2_ − *d*_0,1_ *S*_max⁡_^−1^, the similar method can be used to analyze the sorting rules. However, the following conclusions can be acquired.

- If *td*_1_ − *d*_0,2_ *S*_max⁡_^−1^ ≥ *td*_2_ − *d*_0,1_ *S*_max⁡_^−1^, then *ER*(*SP*_1_) ≤ *ER*(*SP*_2_).
- If *td*_1_ − *d*_0,2_ *S*_max⁡_^−1^ ≤ *td*_2_ − *d*_0,1_ *S*_max⁡_^−1^, then *ER*(*SP*_1_) ≥ *ER*(*SP*_2_).

According to the previous conclusions, the OSR algorithm is proposed to solve the task sorting problem. The main steps of OSR are presented as in Algorithm 1.

The task sorting problem is a combinatorial optimization problem. At present, there is no algorithm which can be used to obtain the optimal solution within the polynomial time complexity. Similar to the EDF algorithm, the OSR algorithm is a heuristic algorithm, which only generates the optimal scheme instead of a common optimized scheme. The effectiveness of the OSR algorithm will be verified in the subsequent experiments.

### 3.2. The VTD Algorithm

According to the task sorting result obtained by OSR, the deadline constraints for each element in set *Task* are checked in order. The value tasks in set *Task* are considered as the key nodes, and the cruising path optimization method between the successive key nodes can be learned from [17, 29, 30].

**Theorem 5**

If both *task*_*i*_ and *task*_*j*_ are value tasks, and *task*_*j*_ is arranged to be executed just next to *task*_*i*_, then *task*_*i*_ is called the preceding task of *task*_*j*_, and *task*_*j*_ is the following task of *task*_*i*_.

Consider *task*_*j*_ to be the following task of *task*_*i*_, so the execution time interval of *task*_*j*_ is presented as follows:

(15) $$\begin{array}{c} tb_{j}=te_{i}+d_{i,j}S_{i,j}^{-1},\\ te_{j}=tb_{j}+t_{j}. \end{array}$$

Assume that the airship has maximal cruising speed while solving the value tasks detection problem based on the hierarchy architecture. The pseudocode of VTD is shown as Algorithm 2.

### 3.3. The EAS Algorithm

If *task*_*i*_ is executed, the power consumption of the high-altitude airship caused by cruising in speed S* to the position *sit*_*j*_ and to observe *task*_*j*_ can be calculated as

(16) energyi,j(S∗)=eni,jactive+enjstatic=αCa[|Wcos⁡(θ2−θ1)|3ti      +di,jS∗|S∗−Wcos⁡(θ2−θ1)|3] +αCn(di,jS∗+ti)|Wsin(θ2−θ1)|3,

where *α* = *ρV* ^2/3^(2*η*)^−1^ is a constant.

If the cruising speed of airship is limited at *S*_*i*,*j*_ ∈ [*S*_min⁡_, *S*_max⁡_], the optimal cruising speed of airship between *sit*_*i*_ and *sit*_*j*_ will be S* = {S* | energy_*i*,*j*_(S*) ≤ energy_*i*,*j*_(*S*), for  all  S*, *S* ∈ [*S*_min⁡_, *S*_max⁡_]}. For the convenience to describe this problem, we define the function of the optimal cruising speed (value range: [*S*_min⁡_, *S*_max⁡_]) between *sit*_*i*_ and *sit*_*j*_ as

(17) $$\begin{array}{c} S^{*}=\text{OPT}{\text{E}}_{i,j}\left(S_{\min},S_{\max}\right). \end{array}$$

**Theorem 6**

If [*S*_1_, *S*_2_]⊃[*S*_3_, *S*_4_], *S*_*a*_ = *OPTE*_*i*,*j*_(*S*_1_, *S*_2_), and *S*_*b*_ = *OPTE*_*i*,*j*_(*S*_3_, *S*_4_), then one can get *en* *e* *r* *g* *y*_*i*,*j*_(*S*_*a*_) ≤ *en* *e* *r* *g* *y*_*i*,*j*_(*S*_*b*_).

**Proof**

Since *S*_*a*_ = OPTE_*i*,*j*_(*S*_1_, *S*_2_), we obtain that energy_*i*,*j*_(*S*_a_) ≤ energy_*i*,*j*_(*S*), for  all  *S* ∈ [*S*_1_, *S*_2_]. For *S*_*b*_ ∈ [*S*_1_, *S*_2_], energy_*i*,*j*_(*S*_*a*_) ≤ energy_*i*,*j*_(*S*_*b*_) exists.

*Inference 1.* If *S*_3_ ∈ [*S*_1_, *S*_2_], *S*_4_ = OPTE_*i*,*j*_(*S*_1_, *S*_2_), then energy_*i*,*j*_(*S*_3_) ≥ energy_*i*,*j*_(*S*_4_) is proven.

**Proof**

It can be considered that *S*_3_ = OPTE_*i*,*j*_(*S*_3_, *S*_3_). According to Theorem 6, since [*S*_3_, *S*_3_]∈[*S*_1_, *S*_2_], so Theorem 6 exists.

Assume that *SP* = 〈*vq*, *sq*〉 is an efficient solution, where *vq* = {*task*_*v*1_, *task*_*v*2_,…, *task*_*vg*_}, and *sq* = {*S*_0,*v*1_, *S*_*v*1,*v*2_,…, *S*_*v*(*g*−1},*vg*_}. Let SP* = 〈*vq*, sq*〉 denote the improved solution of *SP*, and it is obtained by the EAS listed as follows

**Step 1**

Let *task*_0_ denote a virtual task, and set *t*_0_ ← 0, *tb*_0_ ← 0, *tb*_0_ ← 0, *i* ← 0.

**Step 2**

On the premise of ensuring the value tasks, the speed range [*S*_*vi*,*v*(*i*+1)_^min⁡^, *S*_*vi*,*v*(*i*+1)_^min⁡^] of airship cruised from *sit*_*vi*_ to *sit*_*v*(*i*+1)_ is calculated.

**Step 3**

The optimal speed *S*_*vi*,*v*(*i*+1)_* = OPTE(*S*_*vi*,*v*(*i*+1)_^min⁡^, *S*_*vi*,*v*(*i*+1)_^min⁡^) of the airship cruised from *sit*_*vi*_ to *sit*_*v*(*i*+1)_ is calculated by (17). Let *S*_*vi*,*v*(*i*+1)_ ← *S*_*vi*,*v*(*i*+1)_* and *i* ← *i* + 1.

**Step 4**

The execution period of *task*_*vi*_ is updated by (15).

**Step 5**

If *i* ≤ *g*, go to Step 2; otherwise, the iteration ends.

In the previous algorithm, *S*_max⁡_ is the maximum cruising speed of airship along each leg. The minimum cruising speed of airship from *sit*_*vi*_ to *sit*_*v*(*i*+1)_ is

(18) $$\begin{array}{c} S_{vi,v(i+1)}^{\min}=\underset{j=i+1,\ldots ,g}{\max}\left\{\frac{\sum_{k=i}^{j-1}d_{vk,v(k+1)}}{td_{vj}-\sum_{k=i}^{j-1}t_{v(k+1)}-te_{vi}}\right\}. \end{array}$$

**Theorem 7**

As for *task*_*i*_ and *task*_*j*_, if *task*_*i*_ is priority to *task*_*j*_, let *task*_*i*_≻*task*_*j*_ or *task*_*j*_≺*task*_*i*_; otherwise, let *task*_*i*_≺*task*_*j*_ or *task*_*j*_≻*task*_*i*_.

**Theorem 8**

Assume that *SP* = 〈*vq*, *sq*〉 is the optimized solution obtained by the EAS algorithm, and then for all *task*_*vi*_ ∈ *vq* = {*task*_*v*1_, *task*_*v*2_,…, *task*_*vg*_}, *S*_*vi*,*v*(*i*+1)_^min⁡^ ≤ *S*_max⁡_ exists.

**Proof**

For all *task*_*vj*_ ∈ *vq*, *task*_*vj*_≺*task*_*vi*_, since *task*_*vj*_ is a value task, we obtain that

(19) $$\begin{array}{c} te_{vi}+\sum_{k=i}^{j-1}\left(t_{v(k+1)}+d_{vk,v(k+1)}S_{\max}^{-1}\right)\leq td_{vj}. \end{array}$$

We may reach the following conclusion:

(20) $$\begin{array}{c} td_{vj}-\sum_{k=i}^{j-1}t_{v(k+1)}-te_{vi}\geq \sum_{k=i}^{j-1}d_{vk,v(k+1)}S_{\max}^{-1}. \end{array}$$

In other words,

(21) $$\begin{array}{c} \frac{\sum_{k=i}^{j-1}d_{vk,v(k+1)}}{td_{vj}-\sum_{k=i}^{j-1}t_{v(k+1)}-te_{vi}}\leq S_{\max}. \end{array}$$

For the discretion of *task*_*vj*_, we may find that

(22) $$\begin{array}{c} S_{vi,v(i+1)}^{\min}=\underset{j=i+1,\ldots ,g}{\max}\left\{\frac{\sum_{k=i}^{j-1}d_{vk,v(k+1)}}{td_{vj}-\sum_{k=i}^{j-1}t_{v(k+1)}-te_{vi}}\right\}\leq S_{\max}. \end{array}$$

Hereby, Theorem 8 holds.

**Theorem 9**

The optimized solution *SP* = 〈*vq*, *sq*〉 obtained by EAS algorithm is still an efficient solution.

**Proof**

According to Theorem 3, if *SP* = 〈*vq*, *sq*〉 is still an efficient solution, the following conditions are satisfied: (a) for all *task*_*vi*_ ∈ *vq* is a value task; (b) for all *S*_*vi*,*v*(*i*+1)_ ∈ *sq* is no larger than *S*_max⁡_.

As for all *task*_*vi*_ ∈ *vq*, according to (18), we may find that

(23) Svi,v(i+1)min⁡=max⁡j=i+1,…,g{∑k=ij−1dvk,v(k+1)tdvj−∑k=ij−1tv(k+1)−tevi}≥dvi,v(i+1)tdv(i+1)−tv(i+1)−tevi.

In other words,

(24) $$\begin{array}{c} \frac{d_{vi,v(i+1)}}{S_{vi,v(i+1)}^{\min}}\leq td_{v(i+1)}-t_{v(i+1)}-te_{vi}. \end{array}$$

So

(25) $$\begin{array}{c} te_{vi}+\frac{d_{vi,v(i+1)}}{S_{vi,v(i+1)}}+t_{v(i+1)}\leq te_{vi}+\frac{d_{vi,v(i+1)}}{S_{vi,v\left(i+1\right)}^{\min}}+t_{v(i+1)}=td_{v(i+1)}. \end{array}$$

Thus, it can be regarded that *task*_*vi*_ ∈ *vq* is a value task. According to Theorem 8, for all *task*_*vi*_ ∈ *vq*, there always exists *S*_*vi*,*v*(*i*+1)_^min⁡^ ≤ *S*_max⁡_. As for this, there exist *S*_*vi*,*v*(*i*+1)_ ∈ [*S*_*vi*,*v*(*i*+1)_^min⁡^, *S*_max⁡_] and *S*_*vi*,*v*(*i*+1)_ ≤ *S*_max⁡_.

In conclusion, Theorem 9 is valid.

The feasible solution can be converted into an efficient solution by VTD algorithm, so as to figure out the decision variable *X*, *Y*; on this basis, the efficient solution can be transformed into an optimal solution by EAS strategy in order to figure out the decision variable *S*. According to the decision variables, the power consumption of optimized solution *SP* can be described as follows:

(26) $$\begin{array}{c} En_{\text{total}}\left(SP\right)=\sum_{{task}_{i}\in Task}\;\sum_{{task}_{j}\in Task}y_{i,j}{\text{energy}}_{i,j}\left(S_{i,j}\right). \end{array}$$

**Conclusion**

If SP* is the optimized solution of the efficient solution *SP*, there exists *ER*(*SP*) = *ER*(SP*).

**Proof**

According to Theorem 9, SP* is still an efficient solution, so that *SP* and SP* have the same value tasks that is, *ER*(*SP*) = *ER*(SP*) exists.

**Theorem 10**

Assume that *SP* = 〈*vq*, *sq*〉 is an efficient solution and SP* = 〈*vq*, sq*〉 is an optimized solution obtained with EAS algorithm; as for this, SP*⊳*SP* exists.

**Proof**

For any *S*_*vi*,*v*(*i*+1)_* ∈ sq*, there is *S*_*vi*,*v*(*i*+1)_* ∈ [*S*_*vi*,*v*(*i*+1)_^min⁡^, *S*_max⁡_]. According to Theorem 6, energy_*vi*,*v*(*i*+1)_(*S*_*vi*,*v*(*i*+1)_*) ≤ energy_*vi*,*v*(*i*+1)_(*S*_max⁡_) is exists. Therefore, we can get

(27) Entotal(SP∗)=∑taski∈Task ∑taskj∈Taskyi,jenergyi,j(Si,j∗)≤∑taski∈Task ∑taskj∈Taskyi,jenergyi,j(Smax⁡)=Entotal(SP).

Based on Conclusion 1, *ER*(*SP*) = *ER*(SP*); hereby, SP*⊳*SP* exists.

Theorem 10 is actually an authentication to the effectiveness of the EAS algorithm. In other words, if the efficient solution is adjusted in accordance with the EAS algorithm, the obtained result is always more optimal than the original scheme.

## 4. Experimental Analysis

In this section, simulation experiment is conducted to illustrate the effectiveness of the proposed method. For VTD to be the precise algorithm, we only analyze the effectiveness of OSR and EAS.

### 4.1. Experimental Parameters

The proposed algorithms are implemented by Matlab2007 on a PC with Pentium IV 3.06 GHz CPU, 2 GB memory. As far as we know, there are no accepted benchmarks yet in scheduling problem of high-altitude airships, so the random models are used to construct the application scenario and simulate the battlefield area with 200 × 300 km^2^.

The main parameters for high-altitude airships simulation are listed in Table 1.

We divide task number into ten levels from 30 to 300 for offering the flexibility to simulate the various workloads on high-altitude airships. The positions of tasks are generated randomly in battlefield, and their deadline is distributed in a uniform distribution spanning over the active period of airship.

Table 2 gives the configuration of environment parameters employed in our experiment.

Additionally, in order to reduce the calculation complexity of experiments, we let the constant *α* of (16) be equal to “1.” Then the calculation results of total energy consumption are relation values instead of real values.

### 4.2. The Effectiveness of the OSR Algorithm

The heuristic algorithm OSR is employed to solve the task sorting problem. The effectiveness of this algorithm will directly affect the number of value tasks. In order to test the performance of the OSR algorithm, we have compared it with the EDF algorithm and Greedy algorithm.

**Figure 3:**
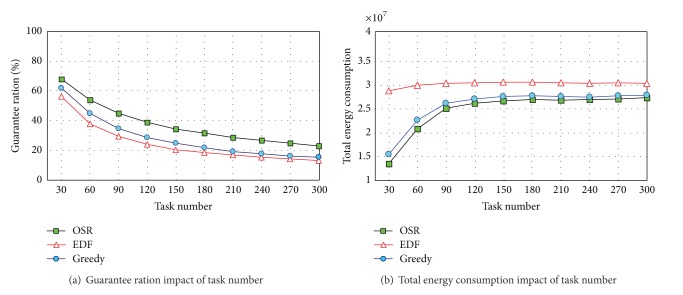
The scheduling results of OSR algorithm.

Figure 3 shows that OSR obtains a higher guarantee ration than EDF and Greedy. In various task scales, the guarantee ration obtained by OSR can be 12.84% higher than that of EDF and 8.89% higher than that of Greedy, which shows a very high scheduling performance. It can be seen in Figure 3(a) that as the quantity of tasks increases, the number of value tasks also increases gradually. However, the task guarantee ration shows a descending tendency. As the quantity of tasks increases, the number of tasks compatible with the airship observation also increases, so that more executable tasks appear. However, the observation capability of airship is limited. Thus, the increasing rate of value task will be much lower than the growth speed of task as the quantity of tasks increases to a certain degree. As for this, the task guarantee ration tends to decrease. As is shown in Figure 3(b), as the task quantity increases, the total energy consumption of airship also increases. However, when the task amount reaches the range of 30~120, the value task amount has a higher growth speed, leading to a sharp increase in energy consumption. By contrast, when task amount is around 120~300, the number of value tasks is nearly saturated, so that the growth of energy consumption slows down.

### 4.3. The Effectiveness of the EAS Algorithm

The EAS algorithm is used to realize energy-saving optimization of the scheduling scheme based on the deadline distribution of value tasks. In order to test the performance of this algorithm, its scheduling results will be compared with the performance before optimization (called HSA algorithm), that is, to compare the optimized solution with the efficient solution. According to Conclusion 1, the optimized solution has the same task guarantee ration as the efficient solution. As for this, the only parameter to be tested is the energy-saving performance of the EAS algorithm. The statistical indexes include the total energy consumption and energy consumption per tasks (ECPT).

**Figure 4:**
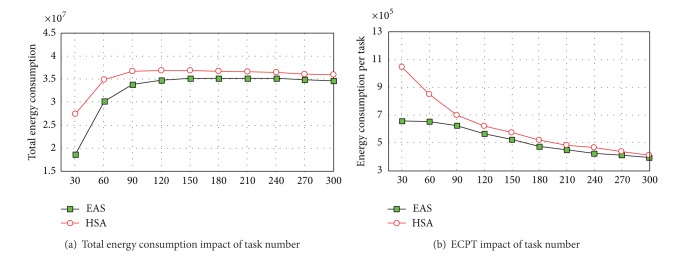
The scheduling results of EAS algorithm.

It can be observed in Figure 4 that the total energy consumption of the EAS algorithm is always lower than that of the HSA algorithm in different task scales. This conclusion complies with Theorem 10. When keeping the same task guarantee ration, the total energy consumption of the EAS algorithm is 8.25% lower than that of the HSA algorithm, showing a higher energy saving performance. It can be seen in Figure 4(a) that the total energy consumption firstly increases sharply and then slows down as the task number increases. This phenomenon is in line with the analysis conclusion in Figure 3(b). The main reason for this trend is the variation tendency of the value task quantity. In Figure 4(b), we may find that ECPT reduces gradually as the task scale increases. As the area of battlefield is a constant, increase of the task scale will improve the density of task in the area. Therefore, the airship is able to execute more tasks within a short route, which in return reduces the energy consumption caused by cruising. In the meantime, we may find that the ECPT of the EAS algorithm is lower than that of the HSA algorithm in different task scales. This is because the EAS algorithm always adopts the most optimal cruising speed in each route, which reduces the energy consumption to the maximum degree.

## 5. Conclusions

The emergency scheduling problem on the high-altitude airship for imaging observation tasks is a multiobjective combination optimization issue. In research, this paper mainly made the following contributions

1. The task execution process of airship is analyzed, and the method to detect the value tasks is provided. In this paper, a power-velocity model was also constructed by considering the influence of the wind field on airship's cruising. Moreover, the programming model of this problem is presented by proposing the optimal objectives and listing corresponding constraints.
2. In order to simplify the solution process, a hierarchy optimization framework which divides the original problem into three subproblems is provided in the paper. As is shown in the experiment, this method is valid in reducing the solution space of the original problem, which is beneficial to efficiently obtain the scheduling scheme.
3. The OSR algorithm is proposed to rank the priority execution order of tasks. The EDF algorithm and Greedy algorithm only use the deadline and cruising distance as the basis of task sorting. By contrast, the OSR algorithm considers both factors at the same time, so as to produce more reasonable results.
4. The EAS algorithm is employed to optimize the scheduling scheme with minimum energy consumption as the objective. This algorithm can adjust the cruising speed of airship in each leg according to the deadline distribution of value tasks. As for this, the total energy consumption of airship can be reduced with no influence on the task guarantee ration. It is of great importance to extend the duration of airship in the observation activity.

Also for our future work, we plan to develop the cooperation scheduling problem of multiple airship in observation activity. This research will be performed based on the research in this paper, with the aim to integrate observation resources and to improve the overall observation efficiency.

## Figures and Tables

**Algorithm 1:**
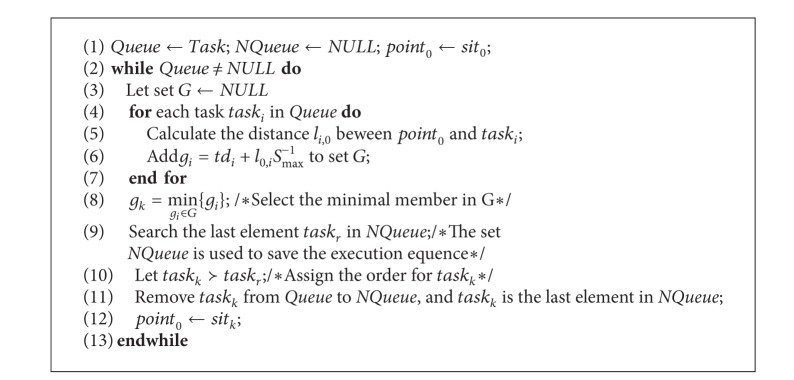
Pseudocode of OSR algorithm.

**Algorithm 2:**
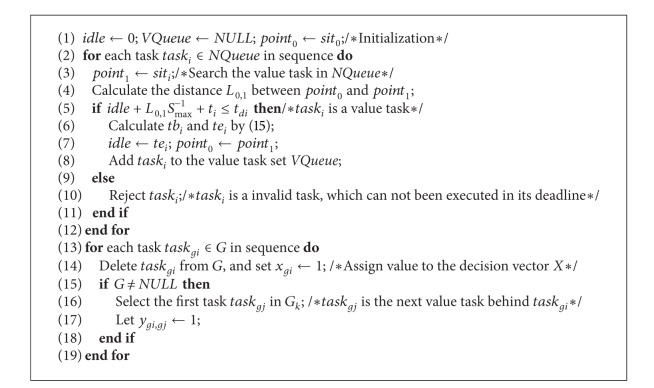
Pseudocode of VTD algorithm.

**Table 1:** Parameters for high-altitude airship.

Parameters	Value
Active period	[6, 18] h
Maximum speed	90 km/h
Efficiency index	0.5
Duration time	[1, 6] min

**Table 2:** Parameters for environment.

Parameters	Value
Wind direction	30°
Wind speed	5 m/s
Aerodynamic coefficient *C*_*a*_	0.025
